# Evaluating displacement of lamina cribrosa following glaucoma surgery

**DOI:** 10.1007/s00417-018-3920-1

**Published:** 2018-02-08

**Authors:** Patrycja Krzyżanowska-Berkowska, Aleksandra Melińska, Iwona Helemejko, D. Robert Iskander

**Affiliations:** 1 Department of Ophthalmology, Wroclaw Medical University, Borowska 213, 50-556 Wroclaw, Poland; 2 Department of Biomedical Engineering, Faculty of Fundamental Problems of Technology, Wroclaw University of Science and Technology, Wybrzeze Wyspianskiego 27, 50-370 Wroclaw, Poland

**Keywords:** Lamina cribrosa, Intraocular pressure, Trabeculectomy, Non-penetrating deep sclerectomy

## Abstract

**Purpose:**

The purpose of the study is to assess the displacement of lamina cribrosa (LC) and prelaminar tissue area (PTA) changes following trabeculectomy and non-penetrating deep sclerectomy (NPDS) using spectral-domain optical coherence tomography (SD-OCT) with enhanced depth imaging technology.

**Methods:**

A total of 30 patients underwent glaucoma surgery. Sixteen patients underwent trabeculectomy, and 14 patients undertook NPDS. Serial horizontal B-scan images of optic nerve head (ONH) were obtained using SD-OCT preoperatively, and at 2-week, 1-, 3-, and 6-month postoperative visit (6 pv). LC displacement magnitude and PTA changes were determined from selected B-scan images. Correspondingly, OCT retinal nerve fiber layer (RNFL) parameters were measured.

**Results:**

Intraocular pressure (IOP) decreased from 27.4 ± 10.3 mmHg (mean ± standard deviation) to 10.2 ± 4.0 mmHg (*P* = 0.011) and from 19.9 ± 4.0 mmHg to 11.9 ± 3.6 mmHg (*P* = 0.012) at 6 pv, for trabeculectomy and NPDS, respectively. There was a significant decrease in the LC depth from a baseline glaucomatous LC displacement of 468.0 ± 142.4 to 397.6 ± 125.2 μm in the trabeculectomy group (*P* = 0.001) and from 465.2 ± 129.6 to 412.0 ± 122.4 μm in the NPDS group (*P* = 0.029) at 6 pv. The PTA differed between the procedures at baseline (*P* = 0.002), but was not statistically significant postoperatively. Multivariate analysis for all patients including age, magnitude of IOP reduction, baseline glaucomatous LC displacement, magnitude of LC displacement, and the type of surgery revealed that only the magnitude of LC displacement was associated with significant RNFL thinning on average (*r*^2^ = 0.162, *P* = 0.027) and in the following sectors: temporal superior (*r*^2^ = 0.197, *P* = 0.014), temporal (*r*^2^ = 0.150, *P* = 0.034), and nasal superior (*r*^2^ = 0.162, *P* = 0.027).

**Conclusions:**

Decrease in the LC depth after NPDS surgery can be observed at 6 pv. Regardless of the performed procedure, magnitude of LC displacement is associated with significant, focal RNFL thinning.

## Introduction

Glaucoma is a chronic, progressive optic neuropathy, in which there is degeneration of retinal ganglion cells with associated gradual loss of visual function. The nerve fibers leave the eyeball through the scleral channel, across which the connective structure is called the lamina cribrosa (LC) [1]. A characteristic feature of glaucomatous optic neuropathy is a pathological enlargement of the cup of the optic disk due to irreversible loss of nerve fibers, and glial cells, and lamina cribrosa distortion [2]. Lowering IOP is currently the only treatment proven effective to prevent disease progression. Surgical IOP reduction causes reversibility of the LC displacement in eyes with primary open angle glaucoma (POAG) [3–5].

The important role of LC structural changes began to surface through post mortem studies of glaucomatous eyes [6, 7] and use of animal models of experimentally induced glaucoma [8, 9]. With the development of the enhanced depth imaging technique using spectral-domain optical coherence tomography, which provides high-resolution images allowing visualization of individual cell layers, changes in the position of the LC and choroid have been studied in vivo. This technique has been used to evaluate the LC in both normal and glaucomatous subjects [10–13].

Lamina cribrosa is a structure that dynamically responds to changes in IOP. Reduction of the LC depth following trabeculectomy was previously described at 6 months and over 2 years postoperatively [3–5]. On the other hand, only one study described changes in the LC position after NPDS [13], and this study highlighted changes in the prelaminar tissue thickness instead of LC position 3 months after surgery. Most NPDS studies were focused on the safety profile, efficacy of IOP reduction, and postoperative complications as compared to other procedures [14–16]. Results that can improve our understanding of the pathophysiology of optic nerve head (ONH) after less invasive procedures are scarce.

The aim of this prospective study was to ascertain whether changes in LC position and the amount of PTA can be observed after NPDS as compared to trabeculectomy up to 6 months postoperatively. Factors were sought that could contribute to the observed changes.

## Materials and methods

### Participants

The study included POAG patients who were followed up for 6 months after surgery. They were enrolled from Glaucoma Clinic at the Department of Ophthalmology, Wroclaw Medical University. The study was approved by the Wroclaw Medical University Review Board and adhered to the tenets of the Declaration of Helsinki. Informed written consent to participate was obtained from all subjects.

All subjects underwent general medical history review and comprehensive ophthalmic examination including refraction, visual acuity measurement, central corneal thickness measurement (PIROP pachymeter, 130909 AP, Echo-Son, Poland), slit-lamp biomicroscopy, Goldmann applanation tonometry, gonioscopy, and dilated examination of the optic disk. Additionally, the retinal nerve fiber layer (RNFL) thickness was measured using the circular scan protocol of the SD-OCT (Spectralis, Heidelberg Engineering GmbH, Heidelberg, Germany). They also underwent standard automated perimetry (Humphrey Field Analyzer II 750; 24-2 SITA-FAST; Carl Zeiss Meditec, Inc., Dublin, CA). A reliable visual field test was defined as one with less than 25% fixation loss and < 30% false positives and negatives.

The inclusion criteria for patients consisted of the following: a diagnosis of POAG, a best corrected visual acuity of ≥ 20/40, spherical refraction of − 3 to + 3 diopters, and cylinder correction within ± 3.0 diopters. POAG was defined as the presence of glaucomatous optic nerve damage (i.e., concentric enlargement of the optic disk, presence of focal thinning, or notching) with associated visual field deterioration in the presence of an open angle. Surgery indication was associated with a confirmed glaucoma progression despite maximally tolerated therapy. Subjects were excluded if they had a history of ocular surgery within 12 months before the onset of the study. Patients with intraocular disease (e.g., diabetic retinopathy, retinal vein occlusion) or neurological disorders affecting visual fields were also excluded from the study.

Patients were scheduled for glaucoma surgery by two of the authors (PK-B and IH). Trabeculectomy was performed with a fornix-based conjunctival flap, rectangular scleral flap, classical iridectomy, and releasable sutures. NPDS was performed with a fornix-based conjunctival flap, rectangular scleral flap, and hyaluronic acid implant (Healaflow™).

### Image acquisition protocol

Serial horizontal B-scan images of the lamina cribrosa were obtained using Spectralis Optical Coherence Tomography (Heidelberg Engineering GmbH, Heidelberg, Germany) preoperatively, 2 weeks, 1, 3, and 6 months postoperatively. The images were gathered using the EDI technique with settings identical to those adopted in other studies [3, 4]. The OCT device was set to image a 15° × 10° vertical rectangle centered on the optic disk. This rectangle was scanned with approximately 75 B-scan section images that were separated by 30 to 34 μm (the scan line distance was determined automatically by the machine). Approximately 42 SD-OCT frames were averaged for each section. This protocol allowed for the best balance between image quality and patient cooperation. The SD-OCT images were acceptable for the study only when the quality score was higher than 18. Images were obtained at 1 day preoperatively and at 2 weeks, 1 month, 3 and 6 months postoperatively. Images of the postoperative period were obtained with the “follow-up” protocol provided by Spectralis OCT, allowing the evaluation of changes at the same location.

### Analysis of the lamina cribrosa depth and prelaminar tissue area

All image processing procedures have been custom written in Matlab (MathWorks, Inc., Natick, MA, USA). Two points characterizing the Bruch’s membrane opening (BMO) and eight points describing the anterior LC were manually marked by an experienced operator (PK-B) for each OCT image using a specially designed graphic user interface. The operator was blinded to the time point of the image. From that, the LC depth was automatically calculated as a maximum perpendicular distance (corresponding to maximally depressed point) between the points of anterior LC surface and the line joining the two points of the BMO, referred further as the BMO line. The mean LC depth was determined by averaging results from 12 to 20 individual central B-scans, where all considered points could be manually annotated without doubt. The number of scans, chosen equidistantly, depended on the size of the optic disk and was selected in a manner to cover up to three quarters of the optic disk [3].

**Fig. 1:**
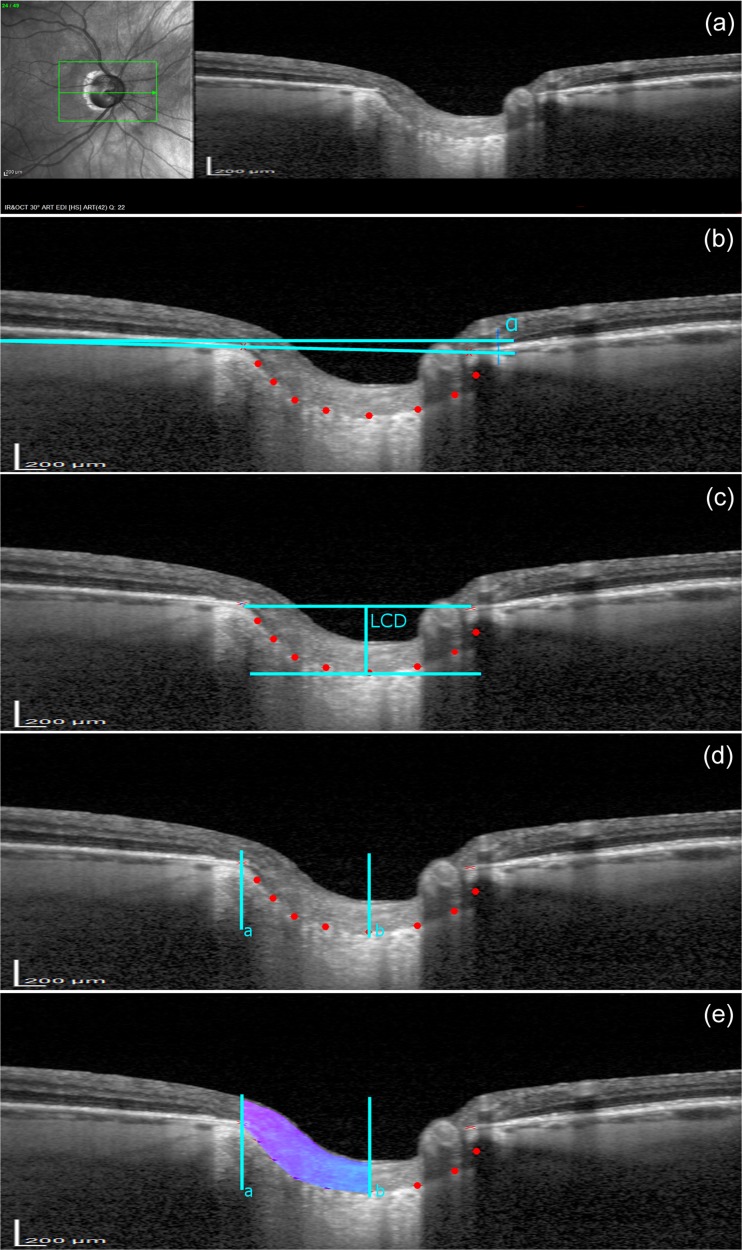
An example of infrared fundus photography and B-scan images obtained at baseline and methods for determination of lamina cribrosa (LC) and prelaminar tissue area (PTA). **a** An example of the acquired OCT image. **b** A reference line at an angle α to the horizontal line was set by connecting two points (red crosses) characterizing the Bruch’s membrane opening (BMO). Eight points describing the anterior LC surface (red dots) were manually placed using a specially designed graphic user interface written in Matlab. **c** An image rotated by α. The LC depth (LCD) was automatically calculated as the maximum perpendicular distance (corresponding to maximally depressed point) between the points of anterior LC surface and the BMO line. **d**, **e** To determine the PTA, the transition between vitreous and the optic cup surface was automatically outlined using image segmentation methods. The prelaminar tissue area was outlined from the top by the optic cup surface, from the bottom by anterior LC, from the temporal side by the line perpendicular to the BMO line (cyan color [a]) and from the nasal side by the line segmenting the LC at maximum LC depth (cyan color [b]), also perpendicular to the BMO line

Prelaminar tissue area (PTA) is an area of soft tissue located between the optic cup surface and the anterior LC surface. To allow estimation of PTA, the transition between vitreous and the optic cup surface was automatically outlined using image segmentation methods. Further, an area outlined: from the top by the optic cup surface, from the bottom by anterior LC, from the temporal side by the line perpendicular to the BMO line, and from the nasal side by the line segmenting the LC at maximum LC depth, also perpendicular to the BMO line. Similarly to LC depth, the mean PTA was determined by averaging results from 12 to 20 individual central B-scans. Figure 1 shows an example of B-scan images with the manually selected points and automatically estimated LC depth and PTA.

Worth noting is that a single pixel of the SD-OCT Spectralis does not correspond to the same vertical and horizontal dimension. Using the scale displaced in the left bottom corner of each SD-OCT image, one can approximate an individual pixel to about 15 μm (horizontal) by 4 μm (vertical) rectangular area. Another issue in assigning the LC depth and PTA physical units is the optical distortion present in all OCT devices and the need to rotate the image as shown in Fig. 1. Hence, in this work, the measured LC depth and PTA are approximately given in units of micrometers and millimeters squared.

### Statistical analysis

Standard descriptive statistics were used along the Wilcoxon and Mann-Whitney tests, which were used to test for differences within and between the groups. Both univariate and multivariate linear regression was used to determine factors associated with IOP reduction, change in visual field parameters, magnitude of LC displacement, and change in the RNFL parameters. For multivariate analyses, a stepwise regression model and a multiple regression model (forward selection and backward elimination) were considered. Statistical analyses were performed using SPSS Statistics, version 22 (SPSS, Inc. Chicago, IL). *P* < 0.05 was considered significant.

## Results

The study included 34 POAG patients aged from 43 to 83 years who were followed for 6 months after surgery. Eighteen patients underwent trabeculectomy and 16 patients non-penetrating deep sclerectomy (NPDS). Of these, 4 patients were lost to follow-up (shortly after surgery); the remaining 16 patients after trabeculectomy and 14 patients after NPDS were followed up at 2 weeks, 1, 3, and 6 months after surgery. The baseline data are presented in the Table 1.

**Table 1:** Baseline data

Baseline variables	Trabeculectomy	NPDS	*P* value^a^
Number of subjects (M/F)	16 (7/9)	14 (9/5)	–
Mean age (years ± SD) (range)	65.4 ± 10.1 (43−79)	66.3 ± 11.4 (53−83)	0.416
Mean CCT (μm ± SD) (range)	531 ± 30 (469−605)	521 ± 30 (431−547)	0.195
Mean AL (mm ± SD) (range)	23.47 ± 1.36 (21.15−25.96)	23.49 ± 0.75 (21.98−24.61)	0.547
Mean IOP (mmHg ± SD) (range)	27.4 ± 10.3 (16−56)	19.9 ± 4.0 (15−26)	*0.006*
Mean VF MD (dB ± SD) (range)	− 15.32 ± 11.03 from − 1.8 to − 30.45	− 15.71 ± 10.99 from − 2.98 to − 28.66	0.462
Mean VF PSD (dB ± SD) (range)	6.23 ± 3.58 (2−13.4)	6.50 ± 2.77 (2.56−13.4)	0.408
Average RNFL thickness (μm ± SD) (range)	55.56 ± 11.93 (37–81)	61.64 ± 19.24 (39–100)	0.159
Number of medications	3.4 ± 0.8 (2−4)	2.9 ± 0.7 (2−4)	0.110

values with statistical significance are shown in italics

*NPDS* non-penetrating deep sclerectomy, *M* male, *F* female, *SD* standard deviation, *CCT* central corneal thickness, *AL* axial length, *IOP* intraocular pressure, *VF MD* visual field mean deviation, *VF PSD* visual field pattern standard deviation, *RNFL* retinal nerve fiber layer

^a^Mann-Whitney test

### Intraocular pressure

In the trabeculectomy group, IOP decreased, on average from 27.4 to 4.4 mmHg at 2 weeks, 7.6 mmHg at 1 month, 9.9 mmHg at 3 months, and 10.2 mmHg at 6 months after surgery. In the NPDS group, IOP decreased from 19.9 to 7.6 mmHg at 2 weeks, 9.6 mmHg at 1 month, 9.7 mmHg at 3 months, and 11.9 mmHg at 6 months after surgery. Statistically significant changes (Mann-Whitney test, *P* < 0.001) were observed between the two considered groups for preoperative levels of IOP. For the group of trabeculectomy, statistically significant reduction of IOP (Wilcoxon test, *P* = 0.011), with respect to the baseline value, has been found at 6 months after surgery, amounting, on average, to 17.3 ± 10.0 mmHg. Similarly, for the group of NPDS, those differences were also significant (*P* = 0.012) and amounted, on average, to 8.0 ± 6.1 mmHg. These amounts correspond to 59.8 ± 19.4 and 37.1 ± 25.9% reduction of IOP in the group of trabeculectomy and NPDS, respectively.

### Factors associated with IOP

**Fig. 2:**
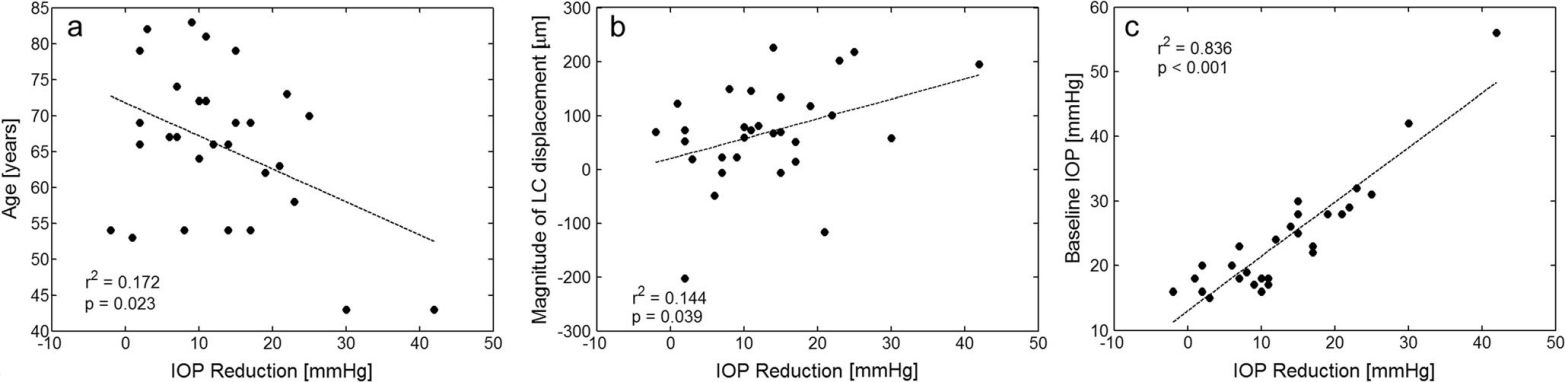
The relationships between IOP reduction at 6 months postoperatively and **a** age, **b** magnitude of LC displacement, and **c** baseline IOP. Data include all patients

Considering all patients as one group, univariate linear regression analysis revealed that the greater IOP reduction at 6 months postoperatively was significantly associated with younger age (*r*^2^ = 0.172, *P* = 0.023), magnitude of LC displacement (*r*^2^ = 0.144, *P* = 0.039), and higher baseline IOP (*r*^2^ = 0.836, *P* < 0.001). The results are presented in Fig. 2.

In the multivariate analysis including age, baseline glaucomatous LC displacement, magnitude of LC displacement, and the type of surgery, the greater IOP reduction was significantly correlated with younger age and the type of surgery (*r*^2^ = 0.400, *P* = 0.001), *P* = 0.013 for age and *P* = 0.004 for the type of surgery.

### Visual field parameters

The visual field parameters mean deviation (MD) and pattern standard deviation (PSD) were measured in both groups before surgery, 1, 3, and 6 months postoperatively. No statistically significant differences were found in visual field parameters between the groups. Considering all patients as one group, statistically significant improvement was found in MD parameter with respect to the baseline value at 1 month (Wilcoxon test, *P* = 0.005), 3 months (*P* = 0.006), and 6 months (*P* = 0.004) postoperatively. No statistically significant changes were found in PSD parameter at 1 month (*P* = 0.329), 3 months (*P* = 0.245), and 6 months (*P* = 0.574) postoperatively. Univariate linear regression analysis revealed no statistically significant correlation between the greater IOP reduction and an improvement of the MD parameter at 6 months postoperatively (*r*^2^ = 0.02, *P* = 0.228). Also, no significant correlation was found between the IOP reduction and PSD parameter at 6 months postoperatively (*r*^2^ = 0.077, *P* = 0.07).

### Retinal nerve fiber layer parameters

**Fig. 3:**
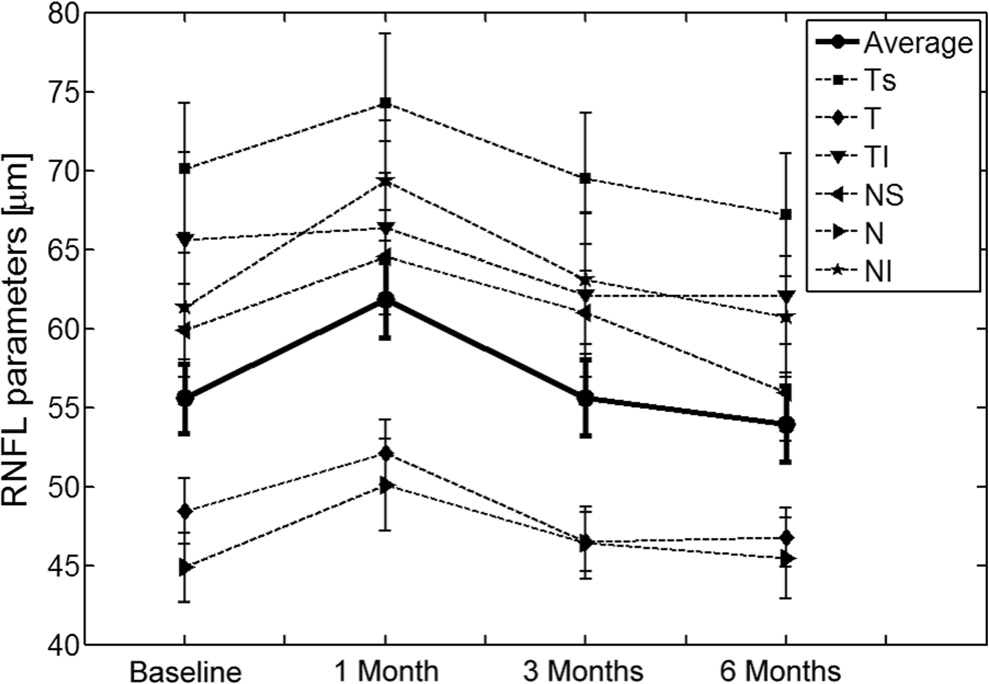
Line graphs showing comparison of the seven parameters of RNFL thickness at each control visit with respect to the baseline values. Bars denote one standard error

Retinal nerve fiber layer thickness (RNFL) was measured by SD-OCT before surgery and at 1, 3, and 6 months postoperatively. No statistically significant differences were found in the global average RNFL thickness neither between the groups (see Table 1) nor the visits. In order to detect localized changes, the following sectors of RNFL were analyzed: temporal superior (TS), temporal (T), temporal inferior (TI), nasal superior (NS), nasal (N), and nasal inferior (NI). For all patients, no statistically significant differences from baseline were found in the following sectors of RNFL: average (Wilcoxon test, *P* = 0.106), T (*P* = 0.244), NS (*P* = 0.909), N (*P* = 0.580), and NI (*P* = 0.216) at 6 months postoperatively. However, statistically significant thinning in TS (from 71.50 ± 27.3 to 68.40 ± 26.9 μm, *P* = 0.018) and TI (from 72.20 ± 32.1 to 67.50 ± 28.3 μm, *P* = 0.047) sectors was found at 6 months postoperatively. The time course of those parameters is depicted in Fig. 3.

**Fig. 4:**
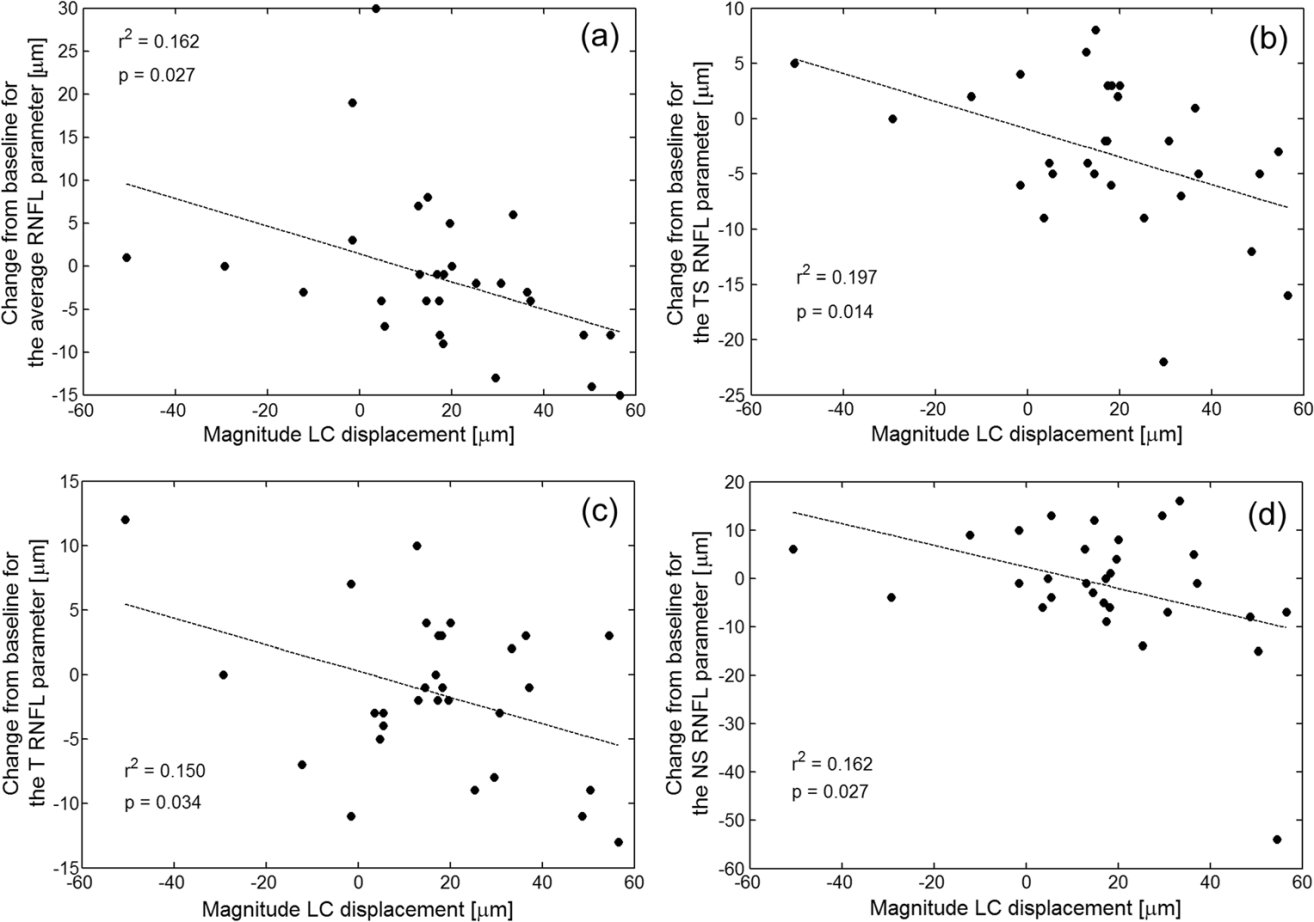
The relationships between the magnitude of LC displacement and the change of RNFL parameters at 6 months postoperatively with respect to the baseline: average RNFL (**a**), TS (**b**), T (**c**), and NS (**d**)

Multivariate analysis for all patients including age, magnitude of IOP reduction, baseline glaucomatous LC displacement, magnitude of LC displacement, and the type of surgery revealed that only the magnitude of LC displacement was associated with significant thinning in the following sectors: average (*r*^2^ = 0.162, *P* = 0.027), TS (*r*^2^ = 0.197, *P* = 0.014), T (*r*^2^ = 0.150, *P* = 0.034), and NS (*r*^2^ = 0.162, *P* = 0.027). The results are presented in Fig. 4. Additionally, univariate linear regression analysis found that the greater IOP reduction was associated with the significant thinning of TS (*r*^2^ = 0.161, *P* = 0.028) and NS (*r*^2^ = 0.145, *P* = 0.038) sectors at 6 months postoperatively.

### Lamina cribrosa and prelaminar tissue area

No statistically significant differences between the groups were found in the LC position before surgery and in the postoperative period (see Table 2). Taking into account the magnitude of the LC anterior displacement within the group, statistically significant changes were observed between the preoperative result and that at each time after surgery (trabeculectomy: *P* = 0.012, *P* < 0.001, *P* = 0.019, and *P* = 0.001; NPDS: *P* = 0.003, *P* = 0.003, *P* = 0.003, and *P* = 0.029; for 2 weeks, 1 month, 3 months, and 6 months postoperatively, respectively).

**Table 2:** Lamina cribrosa depth (maximally depressed point)

Mean LC depth (in micrometers ± SD)	Trabeculectomy	NPDS	*P* value^a^
Preop	468.0 ± 142.4	465.2 ± 129.6	0.480
Postop 2 weeks	398.8 ± 131.2	422.0 ± 134.0	0.324
Postop 1 month	400.0 ± 137.2	406.8 ± 117.2	0.444
Postop 3 months	410.8 ± 122.8	417.2 ± 113.6	0.442
Postop 6 months	397.6 ± 125.2	412.0 ± 122.4	0.383
*P* value^b^ (preop vs. postop 6 months)	*0.001*	*0.029*	

Values with statistical significance are shown in italics

*LC* lamina cribrosa, *SD* standard deviation, *NPDS* non-penetrating deep sclerectomy, *Preop* preoperative, *Postop* postoperative

^a^Mann-Whitney test

^b^Wilcoxon test

**Fig. 5:**
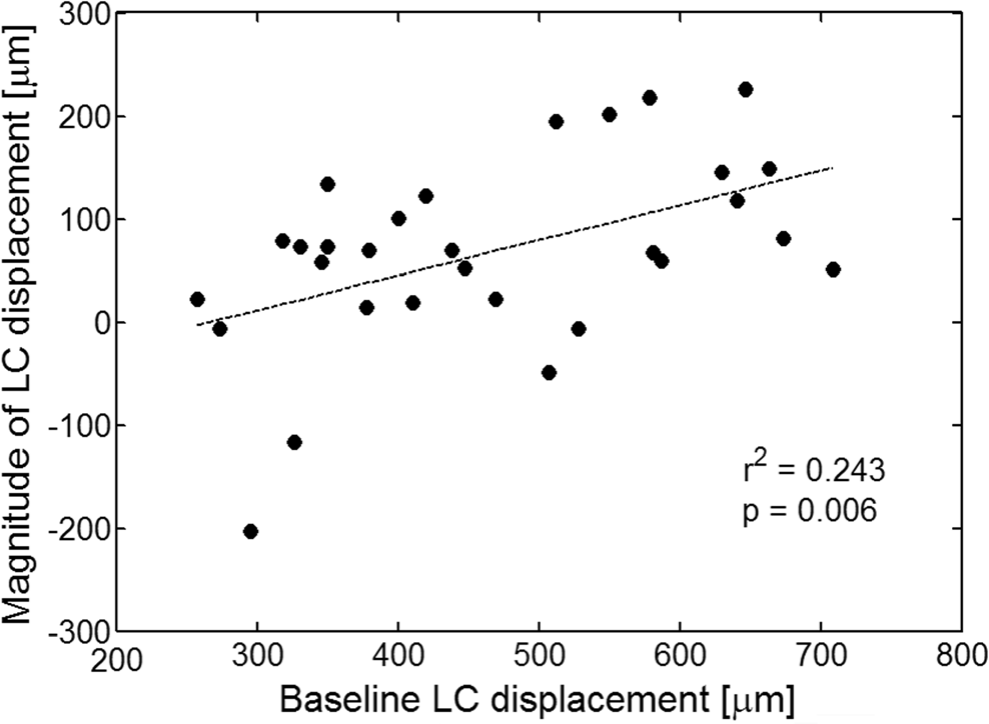
The relationship between the magnitude of LC displacement and the baseline glaucomatous LC displacement. The greater the depth of the LC at the baseline, the more displacement of its position anteriorly towards the vitreous cavity when IOP was reduced

The factors affecting magnitude of the LC displacement were determined for all patients. Multiple linear regression taking into account age, baseline glaucomatous LC displacement, baseline IOP, magnitude of IOP reduction, and the type of surgery revealed that the only factor significantly associated with the magnitude of LC displacement was the baseline glaucomatous LC displacement (*r*^2^ = 0.243, *P* = 0.006). The results are presented in Fig. 5.

In our study, the magnitude of LC displacement did not depend on age (*r*^2^ = 0.062, *P* = 0.185) and the type of surgery (*r*^2^ = 0.073, *P* = 0.362).

The prelaminar tissue area (PTA) differed significantly between the groups preoperatively (0.217 ± 0.094 and 0.331 ± 0.108 mm^2^ for trabeculectomy and NPDS, respectively, Mann-Whitney test, *P* = 0.002). However, in the postoperative period, no statistically significant differences were found in the PTA neither between the groups (*P* = 0.073 at 6 months postoperatively) nor the visits (*P* = 0.145 and *P* = 0.394 for trabeculectomy and NPDS, respectively).

## Discussion

Trabeculectomy effectively decreases IOP as a full-thickness procedure. However, its early postoperative complications are well known. On the contrary, deep sclerectomy is a non-penetrating filtering procedure, which with the adjunctive use of implants, antimetabolites, and goniopuncture may provide final IOP comparable to those obtained with trabeculectomy, but with fewer complications [17, 18].

Our patients were all Caucasians and were matched for age, visual field deterioration, and OCT RNFL parameters. Our results of postoperative IOP reduction in both groups closely correspond to a previous study [14]. Despite differences in IOP when scheduled for the surgery (see Table 1), we observed similar posterior, glaucomatous displacement of the LC preoperatively (468.0 ± 142.4 μm for trabeculectomy and 465.2 ± 129.6 μm for NPDS), and it was interesting to compare whether the type of procedure and the baseline IOP difference can affect the LC displacement results.

One of the main findings of this study was that regardless of the performed procedure, statistically significant anterior displacement of LC was found after substantial magnitude of IOP reduction at 6 months postoperatively. Similarly to Lee et al., who included in their study 12 patients with preoperative IOP within the normal range (18.2 ± 1.9 mmHg) and demonstrated significant reduction in the LC displacement (*P* = 0.002) [3], the present study also found that eyes with lower IOP preoperatively, such as those from the NPDS group, could have statistically significant anterior LC displacement after surgery. This may be relevant for patients scheduled for NPDS who usually have lower IOP at the baseline comparing to patients scheduled for trabeculectomy [18]. Our study also agreed with the results demonstrated previously for the Asian population [3, 5] that the magnitude of the LC displacement was significantly correlated with the baseline glaucomatous LC displacement.

Previous reports described the reversal of optic disk cupping in eyes, in which the surgical IOP reduction was over 30% [19, 20]. In the present study, only one patient in the trabeculectomy group and five patients in the NPDS group had less than 30% of postoperative IOP reduction. Five of those patients (one from the trabeculectomy group and four from the NPDS group) showed a statistically significant LC displacement at 6 months postoperatively. This finding shows that eyes after glaucoma surgery with less than 30% IOP reduction can also achieve the LC displacement. Similar finding was reported previously in a group of newly diagnosed glaucoma subjects and those after trabeculectomy [4].

Considering all patients as one group, our study showed that the greater IOP reduction was significantly correlated with younger age, greater LC displacement, and higher baseline IOP, and these results are in good agreement with other studies [3–5].

Reis et al. reported in 22 glaucoma patients anterior laminar surface displacement and prelaminar tissue thickening in response to IOP decrease at 6 months after glaucoma surgery (18 patients underwent trabeculectomy and four tube shunt implantation) [21]. In comparison to our trabeculectomy group, their group of patients had lower IOP preoperatively (18.1 mmHg), with 33.9% IOP reduction 6 months postoperatively. In contrast to our study, they showed that there was no statistically significant association between the degree of IOP reduction and LC change postoperatively. These discrepancies between the studies confirm the variability of the LC response to IOP reduction and suggest that there are additional factors that determine this response—individual thickness, stiffness, or geometry [22].

One article on the changes in LC after deep sclerectomy that has been published so far is the study by Barrancos et al. [13]. The authors analyzed 28 glaucoma patients, who underwent NPDS and were followed for 3 months postoperatively. Our preoperative IOP measurements of patients scheduled for NPDS (mean IOP 19.9 mmHg) were comparable to the study of Barrancos et al. [13]. However, in postoperative period, our patients presented a greater reduction in pressure comparing to the discussed study (our reduction 37.1% vs. 27.6% of mmHg). This probably explains the difference in lamina cribrosa displacement reported by Barrancos et al., who suggest that early cupping reversal is mainly due to a postoperative increase in the prelaminar tissue (PT) [13]. They explained their results by the gradual not rapid ocular decompression postoperatively and a milder anterior displacement of LC. Because of these results, they decided to finish their follow-up at the third month. Our results however suggest that cupping reversal after NPDS is first of all caused by the anterior movement of LC. Furthermore, this phenomenon cannot be due to increase in PT in our study, because we observed a slight decrease of prelaminar tissue area postoperatively. It is important to note, however, that this decrease was not statistically significant. Hence, it may be more relevant to consider changes in the position of LC with respect to the surgical IOP reduction rather than comparing the type of procedure or baseline IOP.

Numerous studies reported thickening of the prelaminar tissue after lowering the IOP by surgery [3, 4, 13, 21] and thinning after acute IOP elevation [23] in patients with POAG. Based on these studies, it can be suggested that the prelaminar thickness is influenced by IOP; it is compressed when IOP increases and becomes thicker when IOP decreases. However, the prelaminar region comprises many components: bundles of retinal ganglion cell (RGC) axons, astrocytes, capillaries, and extracellular material [24]. Taking into account the differences in tissue thickness depending on the selected measurement position and the presence of blood vessels that hinders its evaluation, we decided that estimates of average thickness do not provide accurate information. Hence, we proposed to outline an area as shown in the Fig. 1 and to compare the mean area obtained during subsequent visits. Based on this protocol, we found that the amount of prelaminar tissue differed significantly between the groups preoperatively and the behavior of the tissue after surgery was also different. The prelaminar tissue area was significantly thinner in the trabeculectomy group compared to the NPDS group (*P* = 0.002), and this could be due to the tissue compression by higher IOP. Postoperatively prelaminar tissue area in the trabeculectomy group became thicker after surgery as in the other studies, while in the NPDS group became thinner after IOP reduction. However, these changes were not statistically significant. It is likely that the mechanism responsible for the prelaminar tissue change after glaucoma surgery is not only an IOP-related factor [25].

Numerous studies evaluated the RNFL thickness after glaucoma surgery [15, 26–29]; fewer studies considered the RNFL thickness and the LC position [4, 13]. The main site of glaucoma damage is believed to be at the lamina cribrosa, though release of pressure on the nerve fibers passing through the lamina should also result in changes in RNFL thickness after IOP reduction. Some studies have shown increased RNFL thickness following glaucoma surgery [27, 29], while other studies [15, 28, 30] showed no change in RNFL thickness on OCT following IOP reduction. However, it is difficult to compare individual studies, due to the large differences in the groups qualified for the study (a combination of POAG and juvenile open-angle glaucoma in one group [27], different baseline values for MD (− 7.0 ± 6.8 dB [15]; MD − 20.4 ± 8.6 dB [29]; − 15.37 ± 10.96 dB in the present study), or analysis of the average RNFL thickness [13] and thickness by quadrants [15, 27, 29].

The present study analyzed thickness of the RNFL on average and in six sectors up to 6 months postoperatively. For all patients, no statistically significant differences were found in the global average RNFL thickness (*P* = 0.106) at 6 months postoperatively. This is consistent with other studies that evaluated RNFL thickness after surgical intervention [12, 15, 27]. Considering the segmental analysis (Fig. 3), statistically significant thinning with respect to the baseline value at 6 months postoperatively was found in two sectors including TS (*P* = 0.018) and TI (*P* = 0.047). This thinning in these two sectors is most likely related to differences in the connective tissue distribution in particular areas of LC. The nasal region of LC has much denser laminar structural tissue with thicker trabeculae than the superior and inferior parts [31]. Progression of the RNFL thinning in the temporal superior and temporal inferior sector despite the IOP reduction may be due to the lack of supportive tissue for axons to maintain their function. It is worth noting that at 6 months postoperatively, the magnitude of LC displacement was statistically significantly associated with thinning of some RNFL areas (see Fig. 4). This phenomenon has not been studied in-depth, and long-term follow-up is needed to verify these results.

This study has some limitations. The sample size in both trabeculectomy and NPDS groups was relatively small. This was a result of subjects being matched for several factors including age, visual field deterioration, and OCT RNFL parameters. Statistical power post hoc estimation was made. The analysis, based on Gaussian assumptions, was conducted for 80% power at the 5% alpha level. For a sample size of 16 subjects in trabeculectomy group and 14 subjects in NPDS group, differences in LC depth of 20 and 19 pixels (corresponding approximately to 80 μm), respectively, could be differentiated. On the other hand, differentiating displacements of about 20 μm would require about 250 subjects at this power level.

Another limitation of this study is the fact that the LC depth was measured from the BMO level, which is influenced by the choroidal thickness. It is well known that the change of the choroidal thickness after surgery would affect the LC depth and may cause the LC depth overestimation postoperatively. However, in our study, the LC depth was still decreased after surgery despite the possibility of overestimation, which reinforces the results of the study. Another approach for measuring the LC depth while avoiding the influence of choroidal thickness is to measure it from the anterior scleral opening level. However, there is no consensus as to whether the anterior scleral opening can be reliably detected on OCT images [5]. Also, the horizontal raster scanning could be improved by considering radial scans which have no limitations to cover the upper and lower parts of the optics disk. Note, however, that in our study, over three quarters of the optic disk was analyzed.

Taking into account the type of surgery, most studies analyzed the change of the LC position after trabeculectomy. There is little data available on other procedures that may affect the LC displacement. It is known that the LC moves anteriorly in response to a significant IOP reduction, as confirmed in this study, but still, we are not able to predict the scope of this movement or the stability of change.

In conclusion, our study showed that regardless of the performed procedure (here trabeculectomy and NPDS), statistically significant anterior displacement of LC takes place after surgical intervention. This displacement was found to be accompanied with localized thinning of RNFL. To the best of our knowledge, there is only one other study considering the changes in LC position after NPDS [13]. Hence, more research is needed to understand the changes in lamina cribrosa position after surgical treatments other than trabeculectomy and the effect of LC displacement on retinal nerve fiber layer.
